# Distributed Bragg Reflectors Employed in Sensors and Filters Based on Cavity-Mode Spectral-Domain Resonances

**DOI:** 10.3390/s22103627

**Published:** 2022-05-10

**Authors:** Michal Gryga, Dalibor Ciprian, Petr Hlubina

**Affiliations:** Department of Physics, Technical University Ostrava, 17. Listopadu 2172/15, 708 00 Ostrava-Poruba, Czech Republic; dalibor.ciprian@vsb.cz

**Keywords:** distributed bragg reflector, band gap, cavity mode, spectral domain, reflectance, transmittance, sensor, filter, sensitivity, figure of merit

## Abstract

Spectral-domain resonances for cavities formed by two distributed Bragg reflectors (DBRs) were analyzed theoretically and experimentally. We model the reflectance and transmittance spectra of the cavity at the normal incidence of light when DBRs are represented by a one-dimensional photonic crystal (1DPhC) comprising six bilayers of TiO${}_{2}$/SiO${}_{2}$ with a termination layer of TiO${}_{2}$. Using a new approach based on the reference reflectance, we model the reflectance ratio as a function of both the cavity thickness and its refractive index (RI) and show that narrow dips within the 1DPhC band gap can easily be resolved. We revealed that the sensitivity and figure of merit (FOM) are as high as 610 nm/RIU and 938 RIU${}^{-1}$, respectively. The transmittance spectra include narrow peaks within the 1DPhC band gap and their amplitude and spacing depend on the cavity’s thickness. We experimentally demonstrated the sensitivity to variations of relative humidity (RH) of moist air and FOM as high as 0.156 nm/%RH and 0.047 %RH${}^{-1}$, respectively. In addition, we show that, due to the transmittance spectra, the DBRs with air cavity can be employed as spectral filters, and this is demonstrated for two LED sources for which their spectra are filtered at wavelengths 680 nm and 780 nm, respectively, to widths as narrow as 2.3 nm. The DBR-based resonators, thus, represent an effective alternative to both sensors and optical filters, with advantages including the normal incidence of light and narrow-spectral-width resonances.

## 1. Introduction

Complex dielectric structures [1] such as one-dimensional photonic crystals (1DPhCs) or distributed Bragg reflectors (DBRs) have attracted enormous interest in research of various topics and applications, such as omnidirectional reflectors [2], polarization selectors [3], optical filters [4,5,6,7], and optical sensors, including Bloch surface wave (BSW) based sensors [8,9,10,11,12,13,14,15,16,17,18,19], Tamm plasmon (TP)-based sensors [20,21,22,23,24,25], gas sensors [15,26,27], and photonic biosensors [28,29,30,31,32,33].

The BSWs are electromagnetic waves that can be localized at the interface between a homogeneous medium to be analyzed and a truncated periodic dielectric medium (1DPhC) decay exponentially along the normal direction, away from the surface into surrounding media. 1DPhCs can be tuned to support BSWs in any desired wavelength range by varying the dispersion and geometry of the periodic dielectric media. Additionally, the integration of PhC technology can further improve light–matter interaction and, thus, the device’s performance [1] due to light guiding and confinement abilities.

1DPhCs exhibit photonic band gaps (PBGs) in which the propagation of electromagnetic waves is forbidden inside the crystal for a specific direction of the incident waves. PBGs can support BSWs for which their excitation is possible by employing a coupling prism to fulfill the resonance condition for the surface wave, in which a strong confinement of light is attained. The excitation of BSWs is similar to that of surface plasmon polaritons (SPPs), but BSWs have several advantages compared to SPPs [34,35]. By suitably changing the geometry and materials of the photonic crystal, BSWs can be excited by both *s*- and *p*-polarized waves [36] at any wavelength, including VIS and NIR regions. Other advantages include high field enhancement leading to high sensitivity, sharper resonances, and higher figure of merits (FOMs) due to absence of metal layer and longer propagation distances [37]. However, a wide range of plasmonic and photonic sensors is available [38,39], including THz ones [40] that outperform BSW-based ones in terms of refractive index sensitivities, figures of merit, and working wavelengths; therefore, they are widely used for refractive index sensing in chemistry, bio-medicine, and the food industry. Moreover, ultrahigh sensitive cavity-coupled plasmonic devices with ultratight confinement of light within the extreme sub-wavelength geometries are available [41].

Most importantly, a direct free-space excitation of BSWs is not possible, contrary to TPs [20], as states of a 1DPhC within the band gap at the interface between a metal and the 1DPhC exist. This concept, which has been extensively studied and utilized [21,42,43], can also be extended to all-dielectric 1DPhC-based sensors employing defects or optical cavities [4,5,6,7,28,29,30,31,32,33,44], characterized by a strong confinement of light in the resonant cavity, which is manifested by very narrow resonances at the normal incidence of light both in transmission and reflection. These resonances exist within the wavelength range of the band gap and correspond to localized modes in the defect, for which its introduction in 1DPhC permits defect states. The position of the defect state in the photonic band gap, or equivalently the resonance wavelength, is given by the refractive index and the thickness of the defect. Thus, optical applications based on 1DPhCs with cavity-mode resonances, especially those experimentally confirmed [45,46,47,48,49,50,51], represent an effective alternative to standard ones.

In this paper, cavities formed by two DBRs and their spectral-domain resonances at the normal incidence of light are analyzed theoretically and experimentally. A new approach based on the reference reflectance of a single DBR from the substrate side is used to model the reflectance ratio as the function of both the cavity thickness and its refractive index. For a 1DPhC comprising six bilayers of TiO${}_{2}$/SiO${}_{2}$ with a termination layer of TiO${}_{2}$, narrow dips within the 1DPhC band gap are resolved, with the sensitivity to refractive index (RI) changes and figure of merit (FOM) reaching 610 nm/RIU and 938 RIU${}^{-1}$, respectively. In addition, the transmittance spectra include narrow peaks within the 1DPhC band gap and their amplitude and spacing depend on the cavity’s thickness. The theoretical results are confirmed by experimental ones, and very high sensitivity and FOM of the narrow dips to variations of relative humidity (RH) of moist air are attained. It is also shown that the DBRs with air cavity can be employed as spectral filters, and this is demonstrated for two LED sources. Thus, the DBR-based resonators represent an effective alternative to both sensors and optical filters employing narrow-spectral-width resonances. Moreover, the dielectric DBRs are mechanically and chemically robust, and they offer the possibility of operation in aggressive environments, with a number of applications for both gaseous and liquid analytes.

## 2. Material Characterization

**Figure 1:**
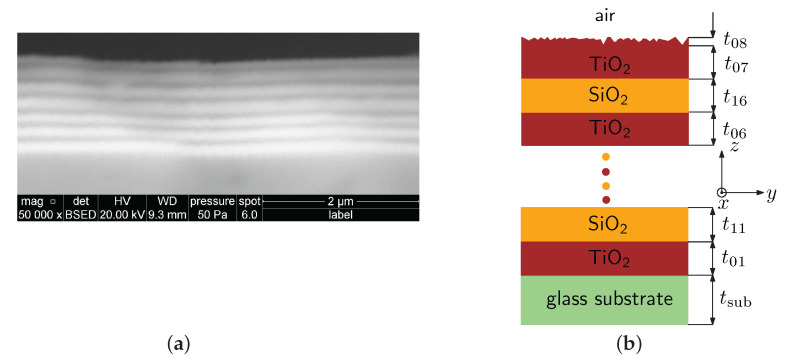
SEM image of a multilayer structure (**a**) and its geometric representation (**b**).

Two DBRs under consideration that form the cavity are represented by a multilayer structure (1DPhC) for which its normal incidence band gap in the reflection spectrum is approximately 280 nm wide (from 590 to 870 nm). 1DPhC was fabricated (Meopta, Přerov, Czech Republic) by a sputtering thin film deposition technique, and exact parameters such as layer thicknesses and dispersions were provided by techniques of scanning electron microscopy (SEM) and spectral ellipsometry. An SEM image (see Figure 1a) captured by a scanning electron microscope (Quanta 650 FEG, FEI Company, Hillsboro, OR, USA) confirmed that the 1DPhC is composed of a system of six bilayers of TiO${}_{2}$/SiO${}_{2}$ and a termination layer of TiO${}_{2}$ demanded for BSW-based sensing applications, as presented in a previous paper [52]. The SEM image obtained for a different magnification can also be found in the same paper. Inspecting the 1DPhC by ellipsometer RC2 (J. A. Woollam Co., Lincoln, NE, USA) and using the variable angle spectroscopic ellipsometry (VASE), VASE data enabled the determination of the thicknesses and refractive index dispersions of the layers employing CompleteEASE software (J. A. Woollam Co.). We have found that six bilayers of TiO${}_{2}$/SiO${}_{2}$ (i=j=1, ..., 6) had thicknesses of $t_{0i}=87.7,79.1,77.3,80.7,80.9$, and 76.9 nm and $t_{1j}=120.2,101.8,109.2,108.0,127.3$, and 125 nm, respectively, and the TiO${}_{2}$ termination layer of thickness $t_{07}=64.4$ nm is has a rough layer of thickness at $t_{08}=7.0$ nm, as schematically shown in Figure 1b. From ellipsometric data, the refractive index dispersion of a glass substrate, TiO${}_{2}$ and SiO${}_{2}$ layers were obtained, as specified in [52].

## 3. Theoretical Analysis

### 3.1. Spectral Reflectance

In the theoretical analysis, the normal incidence of light is considered; to obtain the spectral reflectance of cavity R(λ) formed by two DBRs (1DPhCs), a transfer matrix method (TMM) was used. For the case of *N* dielectric layers, the total transfer matrix M(λ) at the wavelength λ is obtained via transmission matrices across different interfaces and propagation matrices in different homogeneous dielectric media [19]:

(1) $$M\left(\lambda \right)=\left[\begin{array}{cc} M_{11}\left(\lambda \right) & M_{12}\left(\lambda \right)\\ M_{21}\left(\lambda \right) & M_{22}\left(\lambda \right) \end{array}\right]=\left[\prod_{j=1}^{N}B_{j-1,j}\left(\lambda \right)P_{j}\left(\lambda \right)\right]\cdot B_{N,N+1}\left(\lambda \right),$$

where indices 0 and N+1 refer to the first and last semi-infinite media, and $B_{j,j+1}\left(\lambda \right)$ are the boundary matrices respecting the continuity conditions across an interface:

(2) $$B_{j,j+1}\left(\lambda \right)=\frac{1}{2}\left(\begin{array}{cc} 1+\eta (\lambda ) & 1-\eta (\lambda )\\ 1-\eta (\lambda ) & 1+\eta (\lambda ) \end{array}\right),$$

where parameter η(λ) is given by the following:

(3) $$\eta \left(\lambda \right)=\frac{n_{j+1}\left(\lambda \right)}{n_{j}\left(\lambda \right)},$$

with $n_{j}\left(\lambda \right)$ and $n_{j+1}\left(\lambda \right)$ as the refractive indices of media at the interface. Similarly, the propagation matrices are given by the following:

(4) $$P_{j}\left(\lambda \right)=\left(\begin{array}{cc} e^{i\frac{2\pi }{\lambda }n_{j}\left(\lambda \right)t_{j}} & 0\\ 0 & e^{-i\frac{2\pi }{\lambda }n_{j}\left(\lambda \right)t_{j}} \end{array}\right),$$

where $t_{j}$ is the thickness of *j*-th layer.

Spectral reflectance R(λ) is calculated using the total transfer matrix elements as follows.

(5) $$R\left(\lambda \right)={\left|\frac{M_{21}\left(\lambda \right)}{M_{11}\left(\lambda \right)}\right|}^{2}.$$

**Figure 2:**
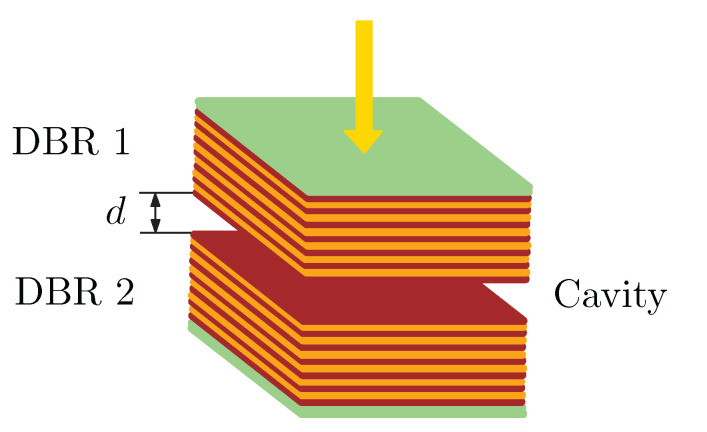
Two DBRs forming cavity of thickness *d*.

To model the reflectance spectrum R(λ) for two DBRs shown in Figure 2 and separated by a cavity thickness of d= 600 nm, we consider that the incident light enters the glass substrate of the first DBR and leaves the glass substrate of the second DBR.

**Figure 3:**
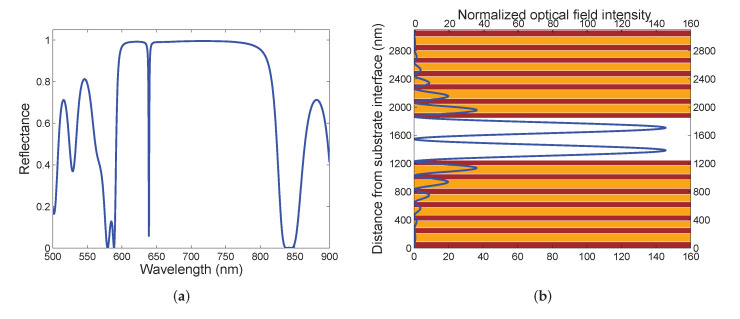
Theoretical spectral reflectance R(λ) at normal incidence for air cavity possesing a thickness of d= 600 nm (**a**). The normalized optical field intensity distribution for the light wave at a wavelength of 639.1 nm (**b**).

The reflectance spectrum R(λ) is computed for air cavity (n=1) using the TMM and taking into account the thicknesses and dispersion of materials of the DBR (1DPhC) specified above and assuming that the extinction coefficients for TiO${}_{2}$ and SiO${}_{2}$ layers are $\kappa_{{\mathrm{TiO}}_{2}}=1.6\times 10^{-3}$ and $\kappa_{{\mathrm{SiO}}_{2}}=3.4\times 10^{-4}$ [19], respectively. As evident from the theoretical spectral reflectance shown Figure 3a, a very narrow dip at a wavelength of 639.1 nm manifests the excitation of the cavity mode. To confirm the cavity mode excitation, the normalized optical field intensity ${\left|E_{x}\right|}^{2}/{\left|E_{x0}\right|}^{2}$ in the structure at the same wavelength is shown in Figure 3b. The computation was performed using the TMM [53] and $E_{x0}$ is *x* component of the electric field of the incident wave, as show in Figure 1b. This figure clearly demonstrates the optical field enhancement in the first DBR, characterized by more than a 145-fold enhancement of the optical intensity with respect to the incident beam and the formation of standing waves between the two DBRs due to the constructive interference of the incident and reflected waves, accompanied by a very narrow dip in the reflectance spectrum.

**Figure 4:**
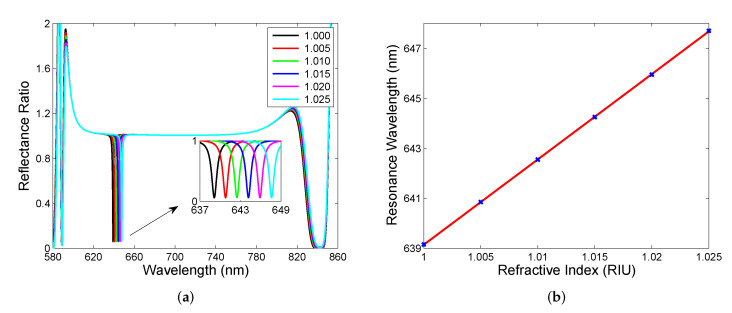
Theoretical spectral reflectance ratio $R\left(\lambda \right)/R_{ref}\left(\lambda \right)$ for cavity having thickness d= 600 nm and analyte with RI in a range of 1.000–1.025 (**a**). Resonance wavelength as a function of the analyte RI (**b**).

Cavity mode undergoing significant change bt varying the cavity’s medium, which has the potential to be used in sensing of various analytes. However, the response can be deteriorated by the source spectrum so it is desirable to use a new approach. The proposed method is based on the reference measurement of the spectral reflectance $R_{ref}\left(\lambda \right)$ of a single DBR from the substrate side so that reflectance ratio $R\left(\lambda \right)/R_{ref}\left(\lambda \right)$ is measured instead of the reflectance spectrum R(λ). To demonstrate the applicability of the approach, Figure 4a shows the theoretical spectral reflectance ratio $R\left(\lambda \right)/R_{ref}\left(\lambda \right)$ for the same cavity thickness and a model gaseous analyte for which its RI changes from 1 to 1.025. In the mentioned reflectance ratio spectrum, sharp and narrow dips within the band gap are apparent, and the wavelength of the dip, the resonance wavelength, is red-shifted as the RI increases. In Figure 4b, the resonance wavelength as a function of the RI is shown together with a linear fitting function, which enables the evaluation of sensitivity to the refractive index $S_{n}$, which is defined as follows:

(6) $$S_{n}=\frac{\delta \lambda_{r}}{\delta n},$$

where $\delta \lambda_{r}$ is the change in the position of the dip with respect to the refractive index change δn of the analyte. In this case, sensitivity $S_{n}$ is constant and reaches 341 nm/RIU. Thus, it is lower than that of simple surface plasmon resonance (SPR) sensors based on the Au layer [39], but it is higher compared to a Tamm plasmon sensor [21]. Among the advantages of the sensing structure, the normal incidence of light operation (no coupling prism is needed) and the ability to be employed in aggressive environments (due to chemical stability of used materials) can be emphasized.

Usually, the performance of the resonance-based sensor is also characterized by a full width at half maximum (FWHM) of the resonance dip or figure of merit (FOM), defined as follows:

(7) $$\mathrm{FOM}=\frac{S_{n}}{\mathrm{FWHM}},$$

and in our case, FOM reaches a value of 341 RIU${}^{-1}$ because of the constant FWHM (1 nm) of the dips. Thus, the cavity-mode resonance-based sensor substantially outperforms the BSW resonance-based ones in FOM, even if the sensitivity is several times smaller [19]. Alternatively, the resonance peak is characterized by the Q-factor, defined as the ratio of the resonance wavelength and FWHM, and in this case, it reaches a value of about 643, which is much higher than those of the SPR [39] and cavity-coupled plasmonic devices [41].

**Figure 5:**
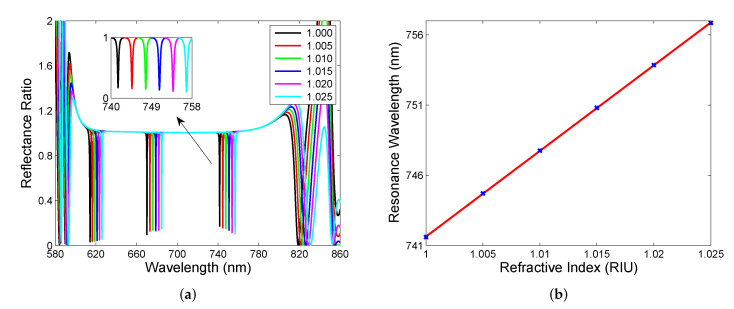
Theoretical spectral reflectance ratio $R\left(\lambda \right)/R_{ref}\left(\lambda \right)$ for cavity having thickness d= 3000 nm and analyte with RI in a range of 1.000–1.025 (**a**). Resonance wavelength as a function of the analyte RI (**b**).

Next, we consider the two DBRs separated by a cavity thickness of d= 3000 nm, and the same gaseous analyte as in the previous case. In Figure 5a, the theoretical spectral reflectance ratio $R\left(\lambda \right)/R_{ref}\left(\lambda \right)$ is shown, and three regions of sharp and narrow resonance dips within the band gap are apparent with the resonance wavelengths red-shifted as RI increases. The excitation of the cavity mode for air at a wavelength of 741.6 nm can be once again confirmed by the normalized optical field intensity similar to that shown in Figure 3b. In this case, nearly a 175-fold field enhancement and eight field peaks in the cavity can be revealed. The resonance wavelength as a function of the RI is shown in Figure 5b for the long-wavelength region with a linear fitting function giving the sensitivity $S_{n}$ as high as 610 nm/RIU. In the remaining regions, sensitivities $S_{n}$ are 466 nm/RIU and 582 nm/RIU, respectively. Because the dips are narrow with a constant FWHM of 0.65 nm, the FOM and Q-factor reach 938 RIU${}^{-1}$ and 1152, respectively. This sensor configuration outperforms available cavity-mode resonance-based sensors [29,30,31,32] in both the sensitivity and FOM. In the reflectance spectrum, the fringes of the highest depth can also be resolved in the spectral region out of the band gap (e.g., near a wavelength of 820 nm), and sensitivities $S_{n}$ are comparable with those of the cavity modes, but FOM is substantially lower due to a greater FWHM.

### 3.2. Spectral Transmittance

Along with the reflectance spectrum, it is crucial to analyze the transmittance spectrum, which is important from the point of view of the spectral filtering. If we assume that the absorption losses of a dielectric multilayer structure are negligible, the spectral transmittance T(λ) can be simply evaluated from the spectral reflectance R(λ) as follows.

(8) T(λ)=1−R(λ).

**Figure 6:**
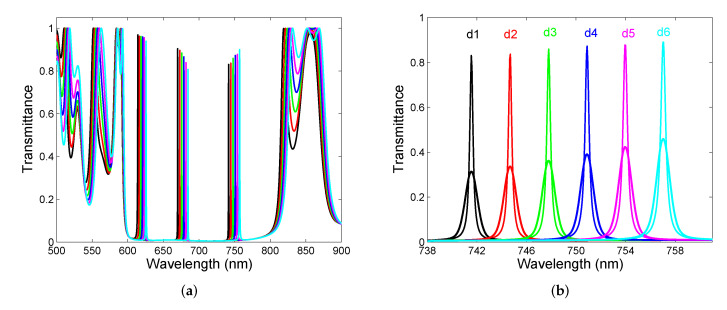
Theoretical spectral transmittance T(λ) for air-cavity thicknesses from $d_{1}=3000$ nm to $d_{6}=3075$ nm (**a**) and a long-wavelength detail including also wider and smaller peaks due to a limited resolving power of a spectrometer (**b**).

As an example, in Figure 6a, the theoretical spectral transmittance T(λ) for different thicknesses of the air cavity formed by the two DBRs is shown, when the cavity thickness is changed from 3000 nm to 3075 nm with a step of 15 nm. Once again, three regions of sharp and narrow resonance peaks within the band gap are revealed with the peak wavelengths red-shifted as the cavity thickness increases. The mutual shift of the resonance peaks is 3.1 nm so that the corresponding sensitivity to the cavity thickness change, or equivalently the change in the position of the resonance peak with respect to the displacement of one of the DBRs, reaches 206 nm/μm. Moreover, the resonance peak amplitude decreases as the wavelength approaches the center of the band gap, which is approximately 710 nm. As can be seen from Figure 6b, narrow peaks have the FWHM of around 0.3 nm so that their actual width cannot be resolved precisely by a standard compact spectrometer. If we suppose that a spectrometer is characterized by a response function $R_{s}\left(\lambda \right)$ of a given spectral bandpass, the transmittance spectrum $T_{s}\left(\lambda \right)$ recorded by the spectrometer can be expressed by the well-known convolution relation [54].

(9) $$T_{s}\left(\lambda \right)=\int T\left(\lambda^{\prime }\right)R_{s}\left(\lambda -\lambda^{\prime }\right)\;d\lambda^{\prime }.$$

The effect of the spectrometer leads to wider and lower peaks, as demonstrated in Figure 6b, when the spectrometer spectral bandpass is supposed to be 1 nm.

## 4. Experimental Setups

**Figure 7:**
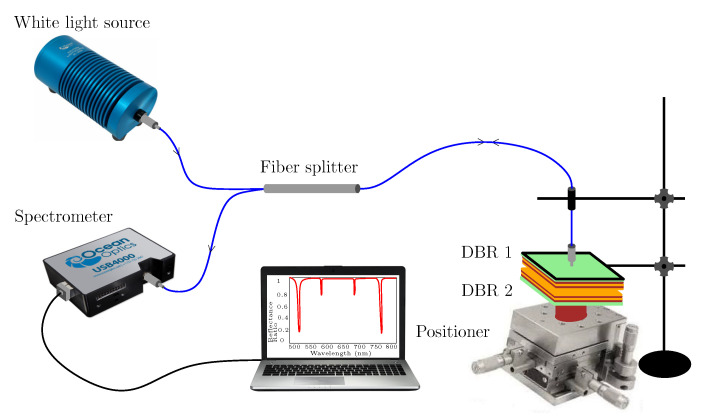
Experimental setup for measuring a reflectance response of two DBRs by employing a multimode fiber splitter.

The spectral reflectance of air cavity formed by two DBRs was measured in two experimental setups, when glue was used to stick the DBR substrate to ring adapter attached to a high resolution translation stage. The first setup is shown in Figure 7 and enables the measurement of spectral reflectance ratio $R\left(\lambda \right)/R_{ref}\left(\lambda \right)$, where reference reflectance $R_{ref}\left(\lambda \right)$ was measured for a single DBR from the substrate side, or equivalently, when DBRs were mutually displaced far away. Light from a white light source (halogen lamp HL-2000, Ocean Optics, Dunedin, FL, USA) is launched into a multimode fiber splitter (SPLIT200-VIS-NIR, Ocean Optics). One arm of the fiber splitter is attached to DBR 1, and DBR 2 is connected to a positioner to adjust cavity thickness. The output arm of the fiber splitter is connected to a compact fiber-optic spectrometer (USB4000, Ocean Optics) to measure the reflectance spectrum in a wavelength range of 400–1000 nm.

**Figure 8:**
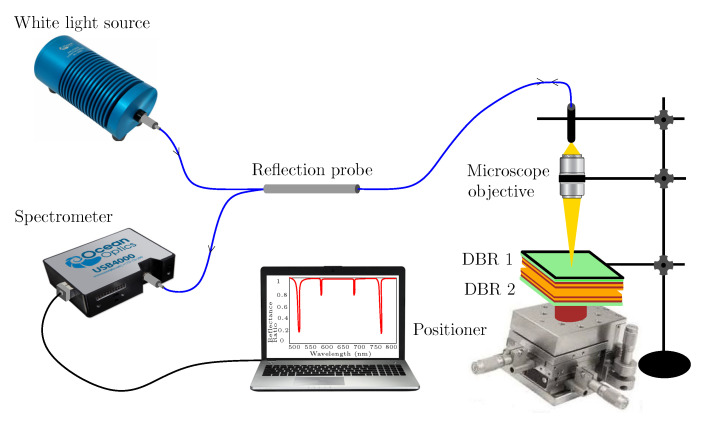
Experimental setup for measuring a reflectance response of two DBRs by employing a reflection probe.

To modify the splitting powers in the reflectance spectrum measurements, a reflection probe (FCR-7IR200-2-ME, Avantes, Apeldoorn, The Netherlands) was used in the second setup shown in Figure 8. A diverging beam of light from the reflection probe is transformed by a microscope objective (10x/0.15, Meopta, Přerov, Czech Republic) into a converging beam of light generating a spot of sub-millimeter diameter on the surface of DBR 1. Thus, a very small area on DBR 1 is illuminated, and the broadening of resonance dips due to lateral inhomogeneities is highly reduced [55].

**Figure 9:**
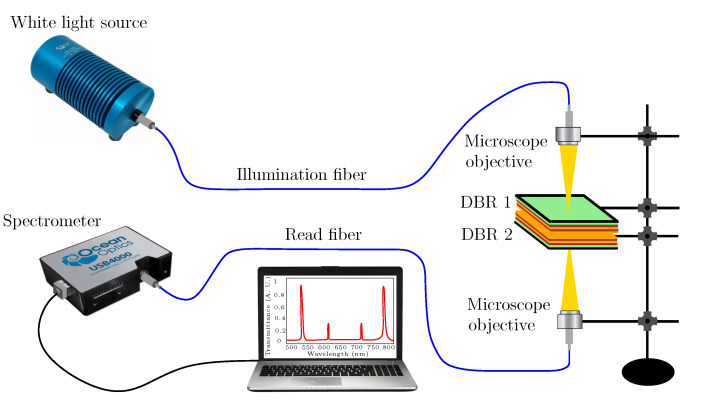
Experimental setup for measuring a transmittance response of two DBRs.

The spectral transmittance of air cavity formed by two DBRs was measured in the experimental setup shown in Figure 9. Light from the white-light source is launched into a coupling fiber terminated by a microscope objective to generate a converging beam of light, which generates a spot of sub-millimeter diameter on the surface of DBR 1. The transmitted light is launched via a microscope objective into a read optical fiber of the compact spectrometer to measure the transmittance spectrum in the wavelength range specified above. The crucial point in all the measurements is to adjust parallelism of both DBRs, which can be controlled by illuminating them by a beam of a sufficiently coherent light and observing interference fringes. Because the substrates are made from a float glass, the interference fringes of various shapes can be revealed.

## 5. Experimental Results and Discussion

**Figure 10:**
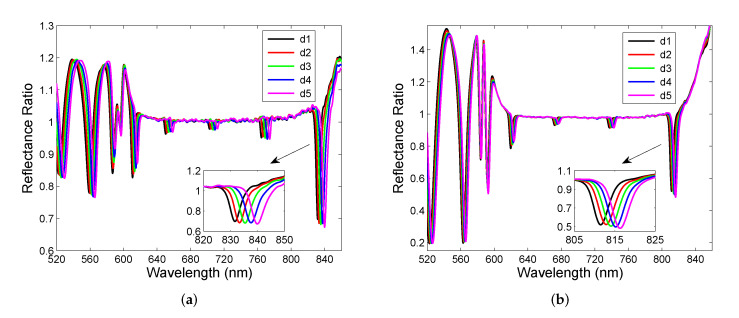
Spectral reflectance ratio $R\left(\lambda \right)/R_{ref}\left(\lambda \right)$ measured for five different cavity thicknesses *d* in the experimental setups shown in Figure 7 (**a**), and Figure 8 (**b**).

In the first step, the experimental setup shown in Figure 7 has been used to measure spectral reflectance ratio $R\left(\lambda \right)/R_{ref}\left(\lambda \right)$ for air cavities formed by two DBRs. The measurements were performed for five different thicknesses of the air cavity when the position of DBR 2 was changed with a step smaller than 500 nm. It should be stressed that this is not actual displacement of the mirror because the substrates are made from a float glass and consequently, and the mirrors may be in contact in some points, as justified by forming a non-uniform interference pattern and hysteresis response. The measurement results depicted in Figure 10a illustrate, in accordance with the theory, that the cavity mode excitations show up as narrow dips with various depths that increase as the dips approach the edges of the band gap. The band gap, which is approximately 280 nm wide (from 590 to 870 nm), is clearly identified from the upper envelope. Out of the band gap are broader spectral fringes (Fabry-Perot resonances) associated with interference of optical waves reflected from the DBRs of the reflectance lower than that within the band gap. The depth of the dips and the contrast of the spectral fringes outside of the band gap are affected by both a fiber core diameter of 200 μm of the splitter and the splitting ratio. The fiber splitter with smaller core diameter and better power splitting could provide higher contrast.

The mutual shift of the resonance dips in a spectral region near a long-wavelength edge of the band gap is nearly constant, as seen from the inset of Figure 10a. In addition, FWHM increases approximately from 4.3 nm to 5.4 nm so that it is substantially smaller than that of the BSW resonances [19]. The FWHM values are affected by both parallelism of the DBRs and a beam spot on the DBR. The parallelism of the DBRs was inspected by an interference pattern for a coherent light source so that the beam spot is possible to adjust close to the zero-order fringe to attain narrowest dips.

To increase both the contrast of the spectral fringes and the depth of resonance dips in the reflectance measurements, the second setup (see Figure 8) employing a reflection probe and a microscope objective has been used. The measurements were performed for five different thicknesses of the air cavity when DBR 2 was displaced with an appropriate step. The measurement results are shown in Figure 10b and illustrate both the contrast and dip depth increase in comparison with the results shown in Figure 10a. The resonance dips near the long-wavelength edge are shown in the inset of Figure 10b, and they are narrow, with FWHM increasing approximately in a range of 4.3–4.9 nm.

**Figure 11:**
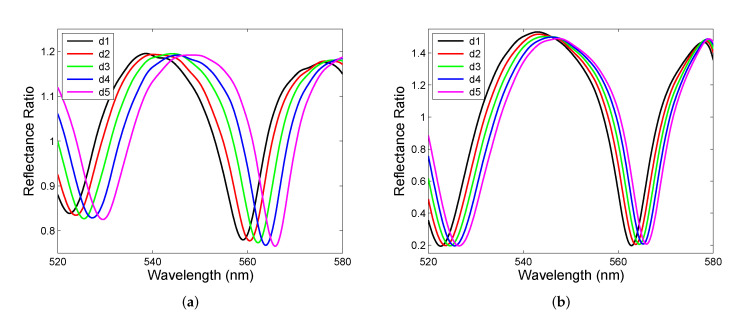
Short-wavelength details of the measured reflectance ratio $R\left(\lambda \right)/R_{ref}\left(\lambda \right)$ from Figure 10a (**a**) and Figure 10b (**b**).

Out of the band gap, the Fabry–Perot resonances are revealed and they show up as spectral interference fringes with the contrast (visibility) dependent on the reflectance measurement technique. The measurement results obtained in the first setup in a short wavelength region are shown Figure 11a. The spectral interference fringes of a sufficiently high contrast are resolved, and to increase contrast, the second setup needs to be used, and the measurement results in a short wavelength region are shown Figure 11b.

**Figure 12:**
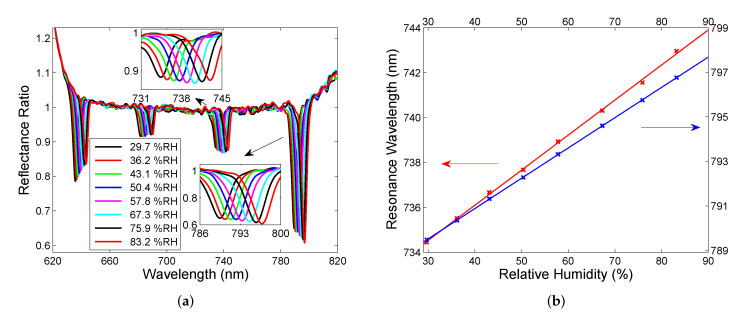
Spectral reflectance ratio $R\left(\lambda \right)/R_{ref}\left(\lambda \right)$ measured for different relative humidities of moist air (**a**), and the corresponding resonance wavelength functions with linear fits (**b**).

In the next step, the first setup (see Figure 7) was used in real RH sensing by employing the cavity formed by two DBRs. In this case, an approach based on using a peristaltic pump and an electrical humidity sensor [35] was adopted to inject moist air of a given RH into the cavity of a suitable thickness and measure the spectral reflectance ratio $R\left(\lambda \right)/R_{ref}\left(\lambda \right)$. The measurement results obtained for a hysteresis-free adjustment with a one substrate side thermally isolated by a thick polystyrene layer are depicted in Figure 12a, and they illustrate that narrow cavity-resonance dips with various depths are red-shifted as the RH of moist air increases. The resonance wavelength as a function of the RH of moist air is shown Figure 12b for two different dips near wavelengths 739 nm and 793 nm, respectively. In both cases, the resonance wavelength shift versus the RH can be well fitted by linear functions so that the sensitivity to RH, defined similarly to Equation (6) as the change of the position of the dip $\delta \lambda_{r}$ with respect to the change in the relative humidity δRH, reaches 0.156 nm/%RH and 0.136 nm/%RH, respectively. Thus, sensing based on the cavity-mode resonances outperforms available techniques [35,52] utilizing surface plasmon and Bloch waves resonances, whispering gallery, and guided mode resonances as well. Using a similar relation to Equation (7), the FOM of the RH measurement reaches 0.047 %RH${}^{-1}$ and 0.036 %RH${}^{-1}$, respectively.

**Figure 13:**
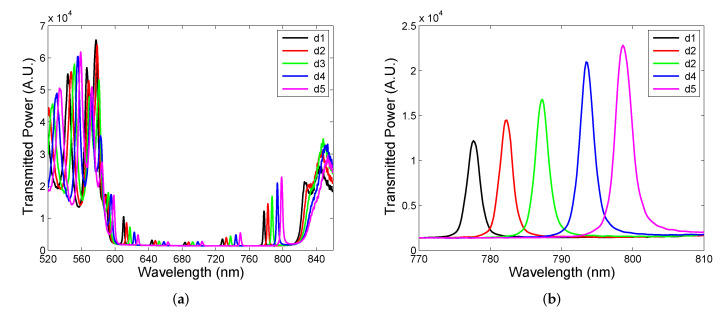
Spectral power transmission measured for five cavity thicknesses *d* changed with a 500 nm step (**a**), and a long-wavelength detail (**b**).

Finally, the spectral power transmitted through the two DBRs with air cavity has been measured in the experimental setup shown in Figure 9. The measurements were performed for five different thicknesses of the cavity when DBR 2 was displaced with a step of approximately 500 nm. The measurement results depicted in Figure 13a illustrate, in accordance with the theory, that cavity mode excitations show up as narrow peaks with various extrema that increase as the peaks approach the edges of the band gap. Out of the band gap, broader spectral fringes are observed and their contrast is lower than one. Higher-contrast fringes and cavity-resonance peaks with a greater amplitude can be attained by a spatial filtering of power detected at the output of the setup.

**Figure 14:**
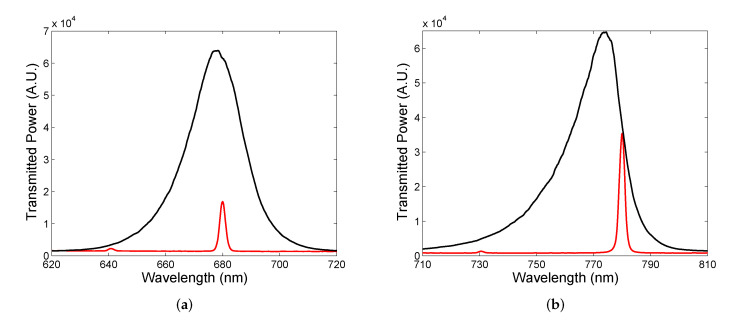
Spectral power transmission measured for an LED alone and combined with a cavity filter: 680 nm (**a**), and 780 nm (**b**).

The resonance peaks near the long-wavelength edge shown in Figure 13b are narrow and the FWHM is approximately in a range of 2.1–2.7 nm. These narrow resonances can be employed in narrow-band filters, and to illustrate the operation of such filters, we utilize two commercially available LEDs of central wavelengths 680 nm (M680F3, Thorlabs, Newton, MA, USA) and 780 nm (M780F2, Thorlabs), respectively. The filtering of the LED spectra employing the DBRs with cavity of an appropriate thickness is illustrated in Figure 14a,b. First, the LED spectrum with the central wavelength of 680 nm and the FWHM of 21.9 nm shown in Figure 14a is filtered to a narrow line with the FWHM of 2.3 nm (Q = 296). Next, the LED spectrum with the central wavelength of 780 nm and the FWHM of 19.9 nm depicted in Figure 14b is filtered to a narrow line with the FWHM of 2.3 nm (Q = 339). Keep in mind that the LED output power is higher when the filter is employed. The relative power of the first light source is lower than that of the second one because the cavity mode resonance in the middle of the band gap is with smaller amplitude than that near the edge of the band gap. Thus, the output power of an LED source employing filter depends on the filter transmittance (see for example Figure 13b). Based on theoretical analysis, the measured FWHMs do not represent actual ones due to the limited resolving power of the spectrometer.

In both filtered spectra, some power is also transmitted by another cavity mode because the LED spectrum is wider than the wavelength spacing (the free spectral range) of the cavity modes. To obtain a single-mode operation of a spectral filter, the cavity thickness needs to be as small as possible in terms of what is attainable by mirrors with substrates of a high flatness, such as λ/10 for BK7 glass substrates (WG11050, Thorlabs) [56]. Generally, output power and the central wavelength of the LED sources with cavity-mode filters can be tuned by a fine adjustment of the cavity’s thickness. In addition, light from an LED source is not absorbed (power of the LED source is not lost) and can be utilized in the reflection of a collimated light by using either a beam splitter between the LED and filter or by employing a oblique incidence of light on the filter.

## 6. Conclusions

In this paper, DBR-based cavity resonances have been analyzed theoretically and experimentally in the spectral-domain. The reflectance and transmittance spectra of the cavity have been modeled at the normal incidence of light, and for DBRs represented by a 1DPhC comprising six bilayers of TiO${}_{2}$/SiO${}_{2}$ with a termination layer of TiO${}_{2}$ and characterized by spectral ellipsometry, narrow dips within the 1DPhC band gap have been revealed. The resonances, simply resolved using a new approach based on the reference reflectance of a single DBR, are sensitive to both the cavity thickness and its refractive index, and the sensitivity and the figure of merit reached 610 nm/RIU and 938 RIU${}^{-1}$, respectively. Similar results have been confirmed for the transmittance spectra that include narrow peaks within the 1DPhC band gap, and their amplitude and spacing depend on the thickness of the cavity.

The theoretical results are confirmed by experimental ones and very high sensitivity and FOM of the narrow dips to variations of RH of moist air, namely 0.156 nm/%RH and 0.047 %RH${}^{-1}$, are attained. In addition, we show that the transmittance spectra for the DBRs with air cavity can be employed as spectral filters, and this is demonstrated for two LED sources for which their spectra are filtered at wavelengths 680 nm and 780 nm, respectively, to widths as narrow as 2.3 nm. The DBR-based resonators with appropriate reflectance or transmittance spectra thus represent an effective alternative to both sensors and optical filters, with advantages including the normal incidence of light and narrow-spectral-width resonances.

The proposed sensing concept, based on the use of the dielectric DBRs that are mechanically and chemically robust, offers the possibility of operation in both VIS and NIR spectral regions [17] and in aggressive environments, with a number of applications for sensing of both gaseous and liquid analytes. The concept can be extended by using cavity formed by an air spacer or a dielectric layer. Moreover, it has advantages in narrow resonance dips or peaks, making the measurement more accurate compared to Fabry–Perot resonance-based sensors. Thus, a great number of new sensors of different quantities and optical filters can be designed and realized.

## Data Availability

Not applicable.
